# Supplementary material for: A high-resolution mRNA expression time course of embryonic development in zebrafish
Source: eLife. 2017 Nov 16;6:e30860. doi: 10.7554/eLife.30860 (PMC5690287; doi:10.7554/eLife.30860)
Supplement: Supplementary file 6. [file elife-30860-supp6.zip › biolayout-clusters-files/Cluster235-genes.html]

Cluster235


# Cluster235: Genes

| Ensembl ID | Gene Name | Chr | Start | End | Biotype |
| --- | --- | --- | --- | --- | --- |
| ENSDARG00000027143 | aptx | 1 | 52018883 | 52024997 | protein\_coding |
| ENSDARG00000101209 | lamb1a | 25 | 31135501 | 31218295 | protein\_coding |
| ENSDARG00000007130 | mrto4 | 11 | 41233822 | 41241747 | protein\_coding |
| ENSDARG00000002487 | noc3l | 12 | 5364723 | 5383383 | protein\_coding |
| ENSDARG00000043304 | nop2 | 16 | 17326563 | 17343921 | protein\_coding |
| ENSDARG00000007744 | tsr1 | 15 | 25700061 | 25721066 | protein\_coding |
